# Ethyl 4-(4-methoxy­phen­yl)-2-oxo-6-phenyl­cyclo­hex-3-ene-1-carboxyl­ate

**DOI:** 10.1107/S1600536808039093

**Published:** 2008-11-26

**Authors:** Hoong-Kun Fun, Samuel Robinson Jebas, Jyothi N. Rao, B. Kalluraya

**Affiliations:** a X-ray Crystallography Unit, School of Physics, Universiti Sains Malaysia, 11800 USM, Penang, Malaysia; b Department of Studies in Chemistry, Mangalore University, Mangalagangothri, Mangalore 574 199, India

## Abstract

The asymmetric unit of the title compound, C_22_H_22_O_4_, consists of two independent mol­ecules (*A* and *B*) which differ significantly in the orientations of ethyl carboxyl­ate groups. The phenyl ring in mol­ecule *B* is disordered over two orientations with occupancies of 0.55 (2) and 0.45 (2). The cyclo­hexenone ring of both mol­ecules adopts an envelope conformation. The dihedral angle between the two aromatic rings is 81.12 (7)° in mol­ecule *A* and 70.8 (3)° in mol­ecule *B* [57.5 (4)° in the minor disorder component]. The crystal structure is stabilized by weak intermolecular C—H⋯O hydrogen bonds and C—H⋯π inter­actions.

## Related literature

For general background, see: Kalluraya & Rai (2003 ▶); Kalluraya & Rahiman (2003 ▶). For bond-length data, see: Allen *et al.* (1987 ▶). For ring puckering analysis, see: Cremer & Pople (1975 ▶).
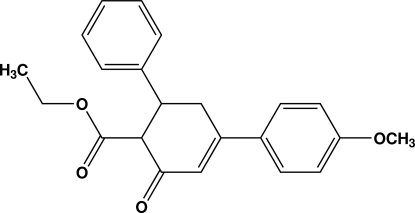


## Experimental

### 

#### Crystal data

C_22_H_22_O_4_
*M* *_r_* = 350.40
Triclinic, 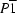

*a* = 9.6095 (2) Å
*b* = 12.5386 (3) Å
*c* = 15.9704 (3) Å
α = 75.916 (1)°
β = 78.879 (1)°
γ = 88.413 (1)°
*V* = 1831.00 (7) Å^3^
*Z* = 4
Mo *K*α radiation
μ = 0.09 mm^−1^
*T* = 100.0 (1) K
0.50 × 0.28 × 0.24 mm

#### Data collection

Bruker SMART APEXII CCD area-detector diffractometer
Absorption correction: multi-scan (*SADABS*; Bruker, 2005 ▶) *T*_min_ = 0.958, *T*_max_ = 0.980
57259 measured reflections
13169 independent reflections
9552 reflections with *I* > 2σ(*I*)
*R*_int_ = 0.032

#### Refinement

*R*[*F* ^2^ > 2σ(*F* ^2^)] = 0.058
*wR*(*F* ^2^) = 0.169
*S* = 1.02
13169 reflections
528 parameters
144 restraints
H-atom parameters constrained
Δρ_max_ = 0.92 e Å^−3^
Δρ_min_ = −0.28 e Å^−3^

### 

Data collection: *APEX2* (Bruker, 2005 ▶); cell refinement: *SAINT* (Bruker, 2005 ▶); data reduction: *SAINT*; program(s) used to solve structure: *SHELXTL* (Sheldrick, 2008 ▶); program(s) used to refine structure: *SHELXTL*; molecular graphics: *SHELXTL*; software used to prepare material for publication: *SHELXTL* and *PLATON* (Spek, 2003 ▶).

## Supplementary Material

Crystal structure: contains datablocks global, I. DOI: 10.1107/S1600536808039093/ci2722sup1.cif

Structure factors: contains datablocks I. DOI: 10.1107/S1600536808039093/ci2722Isup2.hkl

Additional supplementary materials:  crystallographic information; 3D view; checkCIF report

## Figures and Tables

**Table 1:** Hydrogen-bond geometry (Å, °)

*D*—H⋯*A*	*D*—H	H⋯*A*	*D*⋯*A*	*D*—H⋯*A*
C3*A*—H3*AA*⋯O2*B*^i^	1.00	2.57	3.4093 (18)	142
C5*A*—H5*AA*⋯O4*B*^ii^	0.95	2.55	3.3630 (17)	143
C8*A*—H8*AA*⋯O4*B*^ii^	0.95	2.31	3.2446 (18)	166
C14*B*—H14*B*⋯O2*A*^i^	0.95	2.58	3.500 (5)	163
C16*A*—H16*A*⋯O2*B*^iii^	0.95	2.47	3.2789 (18)	143
C17*B*—H17*B*⋯O3*A*^iv^	0.95	2.47	3.116 (9)	125
C21*B*—H21*D*⋯*Cg*1^v^	0.98	2.85	3.811 (2)	167
C22*B*—H22*D*⋯*Cg*2^vi^	0.98	2.87	3.644 (4)	137

Symmetry codes: (i) 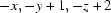; (ii) 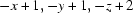; (iii) 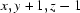; (iv) 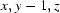; (v) 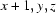; (vi) 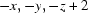. *Cg*1 and *Cg*2 are the centroids of the C13*A*–C18*A* and C13*B*–C18*B* rings, respectively.

**Fig. 1.**
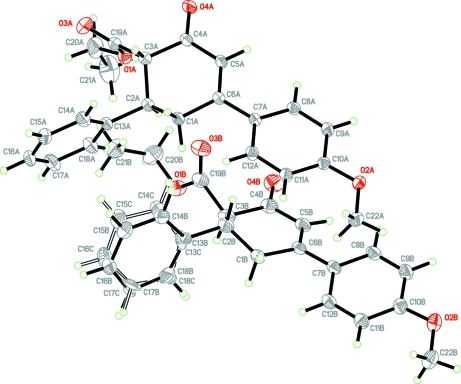
The asymmetric unit of the title compound, showing 50% probability displacement ellipsoids and the atom-numbering scheme. Both disorder component of an independent molecule are shown.

**Fig. 2.**
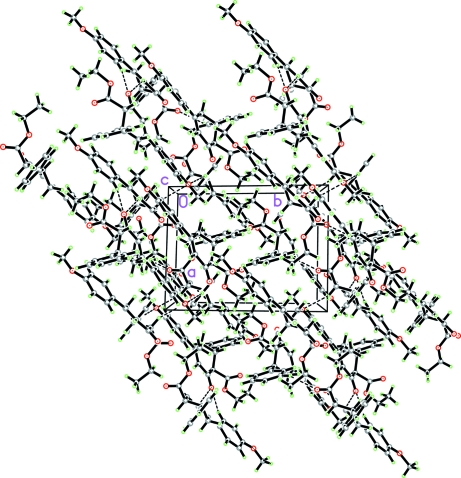
The crystal packing of the title compound, viewed down the c axis. Only the major disorder component of an independent molecule is shown.

## supplementary crystallographic information

### Comment

Cyclohexenone is an organic compound which is a versatile intermediate used in the synthesis of a variety of chemical products such as pharmaceuticals and fragrances. The Robinson annulation is an organic reaction used to create a cyclic six-membered, β-unsaturated ketones. Different methods including solvent-free synthesis of these compounds have been reported (Kalluraya & Rai, 2003). Cyclohexenone and their derivatives are known for anti-inflammatory and analegesic activities (Kalluraya & Rahiman, 2003).

The asymmetric unit of the title compound consists of two independent molecules (Fig. 1). Bond lengths in the molecules (Fig. 1) are found to have normal values (Allen *et al.*, 1987). The two independent molecules differ significantly in the orientations of ethyl carboxylate groups [C4A—C3A—C19A—O1A = 63.32 (14)° and C4B—C3B—C19B—O1B = 105.25 (13)°]. The phenyl ring in molecule B is disordered over two orientations. The cyclohexenone ring in both molecules adopts an envelope conformation, with puckering parameters Q = 0.494 (1) Å, θ = 52.1 (2)° and φ = 67.4 (2)° for molecule A, and Q = 0.429 (2) Å, θ = 130.1 (2)° and φ = 250.4 (3)° in molecule B (Cremer & Pople, 1975). The dihedral angle between the two aromatic rings is 81.12 (7)° in molecule A and 70.8 (3)° in molecule B [57.5 (4)° in the minor disorder component].

The crystal structure is stabilized by intermolecular weak C—H···O hydrogen bonds (Table 1) and C—H···π interactions (Fig 2).

### Experimental

To a solution of 1-phenyl-3-anisyl-prop-2-en-1-one (0.01 mol) in dry acetone (25 ml), dry potassium carbonate (0.04 mol) and ethyl acetoacetate (0.02 mol) in dry acetone (25 ml) were added. The mixture was stirred at room temperature for overnight and was filtered. The solvent from the filtrate on evaporation gave a solid which was recrystallized from a mixture of ethanol-dioxane.

### Refinement

The phenyl ring in molecule B is disordered over two orientations with refined occupancies of 0.545 (17) and 0.455 (17). The C-C bond lengths involving the disordered atoms were restrained to be equal and also the U^ij^ components of the disordered atoms were approximated to isotropic behaviour. The two orientations were restrained to be planar. H atoms were positioned geometrically (C-H = 0.95–1.00 Å) and refined using a riding model, with *U*_iso_(H) = 1.2U_eq_(C) and 1.5U_eq_(C_methyl_). A rotating group model was used for the methyl groups. The highest four difference peaks were observed at 0.92, 0.83, 0.87 and 84 Å, respectively, from atoms C3B, C2B, C2A and C3A. No suitable disorder model involving these atoms were found.

### Crystal data

**Table:**

C_22_H_22_O_4_	*Z* = 4
*M**_r_* = 350.40	*F*_000_ = 744
Triclinic, *P*1	*D*_x_ = 1.271 Mg m^−^^3^
Hall symbol: -P 1	Mo *K*α radiation λ = 0.71073 Å
*a* = 9.6095 (2) Å	Cell parameters from 9088 reflections
*b* = 12.5386 (3) Å	θ = 2.3–33.8º
*c* = 15.9704 (3) Å	µ = 0.09 mm^−^^1^
α = 75.916 (1)º	*T* = 100.0 (1) K
β = 78.879 (1)º	Block, colourless
γ = 88.413 (1)º	0.50 × 0.28 × 0.24 mm
*V* = 1831.00 (7) Å^3^	

### Data collection

**Table:**

Bruker SMART APEXII CCD area-detector diffractometer	13169 independent reflections
Radiation source: fine-focus sealed tube	9552 reflections with *I* > 2σ(*I*)
Monochromator: graphite	*R*_int_ = 0.032
*T* = 100.0(1) K	θ_max_ = 32.5º
φ and ω scans	θ_min_ = 1.9º
Absorption correction: multi-scan(SADABS; Bruker, 2005)	*h* = −14→14
*T*_min_ = 0.958, *T*_max_ = 0.980	*k* = −18→18
57259 measured reflections	*l* = −24→24

### Refinement

**Table:**

Refinement on *F*^2^	Secondary atom site location: difference Fourier map
Least-squares matrix: full	Hydrogen site location: inferred from neighbouring sites
*R*[*F*^2^ > 2σ(*F*^2^)] = 0.058	H-atom parameters constrained
*wR*(*F*^2^) = 0.169	*w* = 1/[σ^2^(*F*_o_^2^) + (0.0817*P*)^2^ + 0.7633*P*] where *P* = (*F*_o_^2^ + 2*F*_c_^2^)/3
*S* = 1.02	(Δ/σ)_max_ = 0.001
13169 reflections	Δρ_max_ = 0.92 e Å^−^^3^
528 parameters	Δρ_min_ = −0.28 e Å^−^^3^
144 restraints	Extinction correction: none
Primary atom site location: structure-invariant direct methods	

### Special details

**Table:**

Experimental. The data was collected with the Oxford Cyrosystem Cobra low-temperature attachment.
Geometry. All esds (except the esd in the dihedral angle between two l.s. planes) are estimated using the full covariance matrix. The cell esds are taken into account individually in the estimation of esds in distances, angles and torsion angles; correlations between esds in cell parameters are only used when they are defined by crystal symmetry. An approximate (isotropic) treatment of cell esds is used for estimating esds involving l.s. planes.
Refinement. Refinement of F^2^ against ALL reflections. The weighted R-factor wR and goodness of fit S are based on F^2^, conventional R-factors R are based on F, with F set to zero for negative F^2^. The threshold expression of F^2^ > 2sigma(F^2^) is used only for calculating R-factors(gt) etc. and is not relevant to the choice of reflections for refinement. R-factors based on F^2^ are statistically about twice as large as those based on F, and R- factors based on ALL data will be even larger.

### Fractional atomic coordinates and isotropic or equivalent isotropic displacement parameters (Å^2^)

**Table:**

	*x*	*y*	*z*	*U*_iso_*/*U*_eq_	Occ. (<1)
O1A	0.40902 (11)	0.86874 (9)	0.67186 (6)	0.0307 (2)	
O2A	−0.31593 (10)	0.42017 (8)	1.15549 (6)	0.02388 (18)	
O3A	0.30572 (13)	1.01757 (9)	0.60083 (7)	0.0387 (3)	
O4A	0.26641 (11)	0.94799 (9)	0.83284 (6)	0.0292 (2)	
C1A	−0.01076 (13)	0.73536 (10)	0.78085 (8)	0.0204 (2)	
H1AA	−0.0901	0.7821	0.7634	0.024*	
H1AB	−0.0321	0.6593	0.7789	0.024*	
C2A	0.12583 (13)	0.77666 (10)	0.71543 (8)	0.0213 (2)	
H2AA	0.2028	0.7253	0.7323	0.026*	
C3A	0.16635 (14)	0.89112 (11)	0.72390 (8)	0.0230 (2)	
H3AA	0.0878	0.9424	0.7101	0.028*	
C4A	0.18266 (13)	0.88549 (10)	0.81804 (8)	0.0222 (2)	
C5A	0.09136 (13)	0.80804 (10)	0.88799 (8)	0.0206 (2)	
H5AA	0.0960	0.8071	0.9471	0.025*	
C6A	−0.00023 (12)	0.73693 (10)	0.87393 (7)	0.0182 (2)	
C7A	−0.08885 (12)	0.65785 (10)	0.94693 (7)	0.0180 (2)	
C8A	−0.05882 (13)	0.63354 (10)	1.03249 (8)	0.0209 (2)	
H8AA	0.0167	0.6718	1.0439	0.025*	
C9A	−0.13674 (13)	0.55552 (11)	1.09986 (8)	0.0220 (2)	
H9AA	−0.1148	0.5408	1.1570	0.026*	
C10A	−0.24827 (12)	0.49764 (10)	1.08465 (8)	0.0190 (2)	
C11A	−0.28153 (13)	0.52069 (11)	1.00134 (8)	0.0214 (2)	
H11A	−0.3580	0.4828	0.9906	0.026*	
C12A	−0.20178 (13)	0.59991 (11)	0.93360 (8)	0.0215 (2)	
H12A	−0.2248	0.6150	0.8767	0.026*	
C13A	0.11848 (14)	0.77910 (11)	0.62083 (8)	0.0224 (2)	
C14A	0.02075 (16)	0.84157 (12)	0.57676 (9)	0.0283 (3)	
H14A	−0.0460	0.8836	0.6066	0.034*	
C15A	0.01971 (17)	0.84323 (12)	0.48886 (9)	0.0314 (3)	
H15A	−0.0477	0.8859	0.4594	0.038*	
C16A	0.11732 (17)	0.78234 (13)	0.44497 (9)	0.0310 (3)	
H16A	0.1164	0.7824	0.3856	0.037*	
C17A	0.21553 (17)	0.72178 (14)	0.48779 (10)	0.0333 (3)	
H17A	0.2833	0.6808	0.4575	0.040*	
C18A	0.21615 (15)	0.72015 (12)	0.57507 (9)	0.0291 (3)	
H18A	0.2846	0.6779	0.6038	0.035*	
C19A	0.30060 (15)	0.93526 (11)	0.65901 (8)	0.0261 (3)	
C20A	0.53784 (18)	0.89483 (17)	0.60642 (11)	0.0431 (4)	
H20A	0.5832	0.9628	0.6106	0.052*	
H20B	0.5168	0.9062	0.5466	0.052*	
C21A	0.6333 (2)	0.7997 (2)	0.62443 (14)	0.0585 (6)	
H21A	0.7196	0.8117	0.5789	0.088*	
H21B	0.5845	0.7322	0.6241	0.088*	
H21C	0.6585	0.7925	0.6821	0.088*	
C22A	−0.42143 (15)	0.35266 (12)	1.14049 (9)	0.0284 (3)	
H22A	−0.4546	0.2953	1.1941	0.043*	
H22B	−0.3806	0.3184	1.0925	0.043*	
H22C	−0.5015	0.3980	1.1245	0.043*	
O1B	0.82102 (11)	0.36693 (9)	0.71886 (8)	0.0337 (2)	
O2B	0.03578 (12)	−0.11336 (9)	1.25183 (7)	0.0331 (2)	
O3B	0.67843 (13)	0.47429 (10)	0.79017 (8)	0.0387 (3)	
O4B	0.77153 (12)	0.27422 (10)	0.92874 (8)	0.0373 (3)	
C1B	0.38209 (13)	0.19261 (11)	0.88538 (8)	0.0232 (2)	
H1BA	0.2799	0.2099	0.8943	0.028*	
H1BB	0.3953	0.1312	0.8558	0.028*	
C2B	0.46513 (14)	0.29275 (12)	0.82477 (9)	0.0260 (3)	
H2BA	0.4347	0.3577	0.8493	0.031*	
C3B	0.62513 (14)	0.27907 (11)	0.82326 (10)	0.0268 (3)	
H3BA	0.6604	0.2205	0.7921	0.032*	
C4B	0.65702 (15)	0.24696 (12)	0.91615 (10)	0.0283 (3)	
C5B	0.55338 (14)	0.18059 (11)	0.98555 (9)	0.0256 (3)	
H5BA	0.5774	0.1538	1.0418	0.031*	
C6B	0.42349 (13)	0.15470 (10)	0.97426 (8)	0.0219 (2)	
C7B	0.32038 (14)	0.08596 (10)	1.04606 (8)	0.0231 (2)	
C8B	0.32756 (16)	0.07361 (12)	1.13529 (9)	0.0281 (3)	
H8BA	0.3999	0.1117	1.1500	0.034*	
C9B	0.23180 (17)	0.00737 (12)	1.20146 (9)	0.0306 (3)	
H9BA	0.2384	0.0006	1.2611	0.037*	
C10B	0.12475 (15)	−0.05007 (11)	1.18154 (9)	0.0263 (3)	
C11B	0.11508 (14)	−0.03927 (12)	1.09398 (9)	0.0262 (3)	
H11B	0.0428	−0.0778	1.0796	0.031*	
C12B	0.21182 (14)	0.02814 (11)	1.02784 (9)	0.0248 (2)	
H12B	0.2042	0.0353	0.9683	0.030*	
C13B	0.4318 (8)	0.3146 (4)	0.7363 (6)	0.0160 (10)	0.545 (17)
C14B	0.3759 (8)	0.4132 (4)	0.6948 (3)	0.0288 (9)	0.545 (17)
H14B	0.3605	0.4707	0.7246	0.035*	0.545 (17)
C15B	0.3424 (9)	0.4304 (4)	0.6127 (3)	0.0367 (11)	0.545 (17)
H15B	0.3050	0.4992	0.5877	0.044*	0.545 (17)
C16B	0.3613 (9)	0.3510 (6)	0.5657 (5)	0.0338 (12)	0.545 (17)
H16B	0.3358	0.3663	0.5094	0.041*	0.545 (17)
C17B	0.4160 (12)	0.2501 (7)	0.5978 (5)	0.0249 (11)	0.545 (17)
H17B	0.4367	0.1944	0.5664	0.030*	0.545 (17)
C18B	0.4363 (14)	0.2418 (9)	0.6820 (6)	0.035 (2)	0.545 (17)
H18B	0.4587	0.1693	0.7101	0.042*	0.545 (17)
C13C	0.4419 (11)	0.3148 (7)	0.7251 (8)	0.033 (2)	0.455 (17)
C14C	0.4258 (11)	0.4248 (6)	0.6876 (4)	0.0334 (13)	0.455 (17)
H14C	0.4284	0.4795	0.7194	0.040*	0.455 (17)
C15C	0.4053 (12)	0.4520 (6)	0.6006 (4)	0.0403 (16)	0.455 (17)
H15C	0.3968	0.5268	0.5711	0.048*	0.455 (17)
C16C	0.3975 (10)	0.3702 (8)	0.5579 (6)	0.0361 (16)	0.455 (17)
H16C	0.3833	0.3855	0.4989	0.043*	0.455 (17)
C17C	0.4117 (16)	0.2646 (9)	0.6059 (7)	0.037 (2)	0.455 (17)
H17C	0.3922	0.2086	0.5788	0.044*	0.455 (17)
C18C	0.4503 (14)	0.2261 (9)	0.6885 (6)	0.0201 (12)	0.455 (17)
H18C	0.4767	0.1535	0.7137	0.024*	0.455 (17)
C19B	0.70784 (15)	0.38573 (12)	0.77640 (10)	0.0288 (3)	
C20B	0.91687 (18)	0.45985 (15)	0.67563 (13)	0.0416 (4)	
H20C	1.0112	0.4325	0.6538	0.050*	
H20D	0.9281	0.5022	0.7189	0.050*	
C21B	0.8642 (2)	0.53375 (16)	0.60068 (12)	0.0456 (4)	
H21D	0.9318	0.5951	0.5732	0.068*	
H21E	0.7718	0.5623	0.6223	0.068*	
H21F	0.8543	0.4924	0.5573	0.068*	
C22B	−0.06909 (17)	−0.18023 (14)	1.23449 (11)	0.0366 (3)	
H22D	−0.1213	−0.2252	1.2902	0.055*	
H22E	−0.0223	−0.2283	1.1980	0.055*	
H22F	−0.1352	−0.1328	1.2034	0.055*	

### Atomic displacement parameters (Å^2^)

**Table:**

	*U*^11^	*U*^22^	*U*^33^	*U*^12^	*U*^13^	*U*^23^
O1A	0.0293 (5)	0.0386 (6)	0.0216 (4)	−0.0049 (4)	−0.0003 (4)	−0.0053 (4)
O2A	0.0278 (4)	0.0217 (4)	0.0209 (4)	−0.0044 (3)	−0.0052 (3)	−0.0017 (3)
O3A	0.0524 (7)	0.0269 (5)	0.0314 (6)	−0.0069 (5)	−0.0031 (5)	0.0001 (4)
O4A	0.0363 (5)	0.0302 (5)	0.0228 (4)	−0.0127 (4)	−0.0039 (4)	−0.0094 (4)
C1A	0.0222 (5)	0.0234 (6)	0.0168 (5)	−0.0032 (4)	−0.0050 (4)	−0.0059 (4)
C2A	0.0248 (5)	0.0220 (5)	0.0178 (5)	−0.0019 (4)	−0.0046 (4)	−0.0057 (4)
C3A	0.0283 (6)	0.0218 (6)	0.0195 (5)	−0.0026 (5)	−0.0042 (4)	−0.0062 (4)
C4A	0.0262 (6)	0.0233 (6)	0.0183 (5)	−0.0033 (5)	−0.0033 (4)	−0.0079 (4)
C5A	0.0233 (5)	0.0238 (6)	0.0156 (5)	−0.0029 (4)	−0.0027 (4)	−0.0067 (4)
C6A	0.0182 (5)	0.0204 (5)	0.0171 (5)	0.0012 (4)	−0.0042 (4)	−0.0061 (4)
C7A	0.0171 (5)	0.0206 (5)	0.0170 (5)	0.0009 (4)	−0.0037 (4)	−0.0056 (4)
C8A	0.0207 (5)	0.0239 (6)	0.0200 (5)	−0.0018 (4)	−0.0066 (4)	−0.0067 (4)
C9A	0.0244 (5)	0.0246 (6)	0.0181 (5)	−0.0004 (4)	−0.0078 (4)	−0.0045 (4)
C10A	0.0197 (5)	0.0179 (5)	0.0192 (5)	0.0014 (4)	−0.0038 (4)	−0.0041 (4)
C11A	0.0193 (5)	0.0248 (6)	0.0210 (5)	−0.0024 (4)	−0.0061 (4)	−0.0049 (4)
C12A	0.0198 (5)	0.0263 (6)	0.0191 (5)	−0.0016 (4)	−0.0061 (4)	−0.0046 (4)
C13A	0.0278 (6)	0.0233 (6)	0.0168 (5)	−0.0059 (5)	−0.0049 (4)	−0.0051 (4)
C14A	0.0340 (7)	0.0278 (6)	0.0248 (6)	0.0019 (5)	−0.0084 (5)	−0.0078 (5)
C15A	0.0421 (8)	0.0286 (7)	0.0237 (6)	−0.0020 (6)	−0.0129 (6)	−0.0015 (5)
C16A	0.0419 (8)	0.0354 (7)	0.0163 (5)	−0.0095 (6)	−0.0061 (5)	−0.0055 (5)
C17A	0.0356 (7)	0.0424 (8)	0.0255 (6)	−0.0003 (6)	−0.0043 (5)	−0.0164 (6)
C18A	0.0326 (7)	0.0345 (7)	0.0243 (6)	0.0012 (6)	−0.0094 (5)	−0.0118 (5)
C19A	0.0341 (7)	0.0251 (6)	0.0190 (5)	−0.0091 (5)	−0.0016 (5)	−0.0067 (5)
C20A	0.0326 (7)	0.0604 (11)	0.0305 (8)	−0.0117 (7)	0.0078 (6)	−0.0094 (7)
C21A	0.0303 (8)	0.0938 (17)	0.0459 (11)	0.0043 (9)	0.0024 (7)	−0.0146 (11)
C22A	0.0315 (6)	0.0246 (6)	0.0281 (6)	−0.0078 (5)	−0.0072 (5)	−0.0025 (5)
O1B	0.0325 (5)	0.0284 (5)	0.0403 (6)	−0.0011 (4)	−0.0040 (4)	−0.0110 (4)
O2B	0.0379 (5)	0.0327 (5)	0.0260 (5)	0.0015 (4)	−0.0083 (4)	−0.0003 (4)
O3B	0.0427 (6)	0.0348 (6)	0.0435 (6)	0.0031 (5)	−0.0090 (5)	−0.0181 (5)
O4B	0.0319 (5)	0.0417 (6)	0.0440 (6)	−0.0052 (5)	−0.0215 (5)	−0.0093 (5)
C1B	0.0226 (5)	0.0267 (6)	0.0234 (6)	0.0011 (5)	−0.0094 (4)	−0.0083 (5)
C2B	0.0253 (6)	0.0276 (6)	0.0289 (6)	0.0023 (5)	−0.0093 (5)	−0.0109 (5)
C3B	0.0252 (6)	0.0259 (6)	0.0338 (7)	0.0018 (5)	−0.0103 (5)	−0.0124 (5)
C4B	0.0260 (6)	0.0295 (7)	0.0344 (7)	0.0026 (5)	−0.0146 (5)	−0.0108 (5)
C5B	0.0294 (6)	0.0249 (6)	0.0280 (6)	0.0044 (5)	−0.0144 (5)	−0.0105 (5)
C6B	0.0257 (6)	0.0210 (5)	0.0234 (6)	0.0051 (4)	−0.0096 (4)	−0.0101 (4)
C7B	0.0279 (6)	0.0220 (6)	0.0232 (6)	0.0055 (5)	−0.0097 (5)	−0.0093 (5)
C8B	0.0376 (7)	0.0260 (6)	0.0254 (6)	0.0020 (5)	−0.0142 (5)	−0.0088 (5)
C9B	0.0426 (8)	0.0284 (7)	0.0236 (6)	0.0045 (6)	−0.0137 (6)	−0.0067 (5)
C10B	0.0305 (6)	0.0247 (6)	0.0237 (6)	0.0080 (5)	−0.0079 (5)	−0.0046 (5)
C11B	0.0258 (6)	0.0289 (6)	0.0263 (6)	0.0039 (5)	−0.0083 (5)	−0.0088 (5)
C12B	0.0259 (6)	0.0285 (6)	0.0230 (6)	0.0041 (5)	−0.0089 (5)	−0.0093 (5)
C13B	0.0167 (15)	0.0147 (16)	0.017 (2)	0.0025 (12)	−0.0014 (12)	−0.0057 (12)
C14B	0.031 (2)	0.0253 (15)	0.0305 (15)	−0.0005 (16)	−0.0052 (16)	−0.0084 (11)
C15B	0.049 (3)	0.0263 (16)	0.0327 (17)	0.0033 (17)	−0.0118 (18)	−0.0008 (12)
C16B	0.043 (3)	0.036 (2)	0.0238 (19)	−0.005 (2)	−0.013 (2)	−0.0042 (15)
C17B	0.042 (2)	0.0218 (18)	0.0146 (16)	−0.0060 (16)	−0.0051 (15)	−0.0108 (18)
C18B	0.049 (4)	0.018 (3)	0.035 (3)	0.001 (2)	−0.014 (2)	0.0042 (18)
C13C	0.030 (3)	0.055 (4)	0.015 (3)	−0.017 (2)	−0.008 (2)	−0.003 (2)
C14C	0.040 (3)	0.035 (2)	0.028 (2)	0.006 (2)	−0.011 (2)	−0.0114 (17)
C15C	0.054 (4)	0.035 (2)	0.031 (2)	0.014 (3)	−0.012 (2)	−0.0056 (17)
C16C	0.033 (3)	0.050 (4)	0.026 (2)	0.005 (2)	−0.012 (2)	−0.006 (3)
C17C	0.045 (3)	0.041 (4)	0.032 (3)	−0.016 (3)	0.002 (2)	−0.028 (2)
C18C	0.033 (2)	0.012 (3)	0.017 (2)	0.0041 (19)	−0.0100 (16)	−0.0024 (19)
C19B	0.0255 (6)	0.0328 (7)	0.0323 (7)	−0.0010 (5)	−0.0109 (5)	−0.0116 (6)
C20B	0.0308 (7)	0.0402 (9)	0.0492 (10)	−0.0045 (6)	−0.0038 (7)	−0.0047 (7)
C21B	0.0490 (10)	0.0414 (9)	0.0433 (9)	−0.0057 (8)	−0.0069 (8)	−0.0050 (7)
C22B	0.0342 (7)	0.0356 (8)	0.0353 (8)	−0.0006 (6)	−0.0090 (6)	0.0021 (6)

### Geometric parameters (Å, °)

**Table:**

O1A—C19A	1.3320 (18)	C1B—C6B	1.5092 (17)
O1A—C20A	1.4452 (18)	C1B—C2B	1.5220 (19)
O2A—C10A	1.3648 (15)	C1B—H1BA	0.99
O2A—C22A	1.4289 (16)	C1B—H1BB	0.99
O3A—C19A	1.2038 (17)	C2B—C13B	1.468 (8)
O4A—C4A	1.2313 (15)	C2B—C3B	1.5385 (19)
C1A—C6A	1.5143 (16)	C2B—C13C	1.607 (12)
C1A—C2A	1.5257 (17)	C2B—H2BA	1.00
C1A—H1AA	0.99	C3B—C19B	1.524 (2)
C1A—H1AB	0.99	C3B—C4B	1.528 (2)
C2A—C13A	1.5189 (16)	C3B—H3BA	1.00
C2A—C3A	1.5412 (17)	C4B—C5B	1.444 (2)
C2A—H2AA	1.00	C5B—C6B	1.3541 (18)
C3A—C19A	1.5131 (18)	C5B—H5BA	0.95
C3A—C4A	1.5259 (17)	C6B—C7B	1.4723 (19)
C3A—H3AA	1.00	C7B—C12B	1.4000 (18)
C4A—C5A	1.4498 (17)	C7B—C8B	1.4104 (18)
C5A—C6A	1.3554 (16)	C8B—C9B	1.376 (2)
C5A—H5AA	0.95	C8B—H8BA	0.95
C6A—C7A	1.4726 (16)	C9B—C10B	1.399 (2)
C7A—C12A	1.3997 (16)	C9B—H9BA	0.95
C7A—C8A	1.4099 (16)	C10B—C11B	1.3927 (18)
C8A—C9A	1.3757 (18)	C11B—C12B	1.387 (2)
C8A—H8AA	0.95	C11B—H11B	0.95
C9A—C10A	1.4013 (17)	C12B—H12B	0.95
C9A—H9AA	0.95	C13B—C18B	1.398 (8)
C10A—C11A	1.3884 (16)	C13B—C14B	1.399 (6)
C11A—C12A	1.3941 (17)	C14B—C15B	1.375 (6)
C11A—H11A	0.95	C14B—H14B	0.95
C12A—H12A	0.95	C15B—C16B	1.373 (7)
C13A—C18A	1.3861 (19)	C15B—H15B	0.95
C13A—C14A	1.3917 (19)	C16B—C17B	1.376 (8)
C14A—C15A	1.4008 (19)	C16B—H16B	0.95
C14A—H14A	0.95	C17B—C18B	1.376 (8)
C15A—C16A	1.386 (2)	C17B—H17B	0.95
C15A—H15A	0.95	C18B—H18B	0.95
C16A—C17A	1.375 (2)	C13C—C18C	1.371 (9)
C16A—H16A	0.95	C13C—C14C	1.381 (9)
C17A—C18A	1.3903 (19)	C14C—C15C	1.399 (7)
C17A—H17A	0.95	C14C—H14C	0.95
C18A—H18A	0.95	C15C—C16C	1.374 (8)
C20A—C21A	1.491 (3)	C15C—H15C	0.95
C20A—H20A	0.99	C16C—C17C	1.377 (10)
C20A—H20B	0.99	C16C—H16C	0.95
C21A—H21A	0.98	C17C—C18C	1.407 (10)
C21A—H21B	0.98	C17C—H17C	0.95
C21A—H21C	0.98	C18C—H18C	0.95
C22A—H22A	0.98	C20B—C21B	1.488 (3)
C22A—H22B	0.98	C20B—H20C	0.99
C22A—H22C	0.98	C20B—H20D	0.99
O1B—C19B	1.3373 (18)	C21B—H21D	0.98
O1B—C20B	1.451 (2)	C21B—H21E	0.98
O2B—C10B	1.3619 (18)	C21B—H21F	0.98
O2B—C22B	1.436 (2)	C22B—H22D	0.98
O3B—C19B	1.1995 (18)	C22B—H22E	0.98
O4B—C4B	1.2279 (16)	C22B—H22F	0.98
			
C19A—O1A—C20A	116.22 (12)	C13B—C2B—C13C	4.7 (6)
C10A—O2A—C22A	117.00 (10)	C1B—C2B—C13C	113.0 (4)
C6A—C1A—C2A	112.11 (9)	C3B—C2B—C13C	107.6 (4)
C6A—C1A—H1AA	109.2	C13B—C2B—H2BA	107.6
C2A—C1A—H1AA	109.2	C1B—C2B—H2BA	107.6
C6A—C1A—H1AB	109.2	C3B—C2B—H2BA	107.6
C2A—C1A—H1AB	109.2	C13C—C2B—H2BA	110.3
H1AA—C1A—H1AB	107.9	C19B—C3B—C4B	106.55 (11)
C13A—C2A—C1A	113.81 (10)	C19B—C3B—C2B	111.56 (11)
C13A—C2A—C3A	110.83 (10)	C4B—C3B—C2B	111.59 (12)
C1A—C2A—C3A	109.34 (10)	C19B—C3B—H3BA	109.0
C13A—C2A—H2AA	107.5	C4B—C3B—H3BA	109.0
C1A—C2A—H2AA	107.5	C2B—C3B—H3BA	109.0
C3A—C2A—H2AA	107.5	O4B—C4B—C5B	122.18 (13)
C19A—C3A—C4A	111.12 (10)	O4B—C4B—C3B	119.32 (14)
C19A—C3A—C2A	110.74 (10)	C5B—C4B—C3B	118.42 (11)
C4A—C3A—C2A	110.11 (10)	C6B—C5B—C4B	123.11 (12)
C19A—C3A—H3AA	108.3	C6B—C5B—H5BA	118.4
C4A—C3A—H3AA	108.3	C4B—C5B—H5BA	118.4
C2A—C3A—H3AA	108.3	C5B—C6B—C7B	121.91 (11)
O4A—C4A—C5A	122.27 (11)	C5B—C6B—C1B	120.34 (12)
O4A—C4A—C3A	120.41 (11)	C7B—C6B—C1B	117.70 (11)
C5A—C4A—C3A	117.25 (10)	C12B—C7B—C8B	117.19 (12)
C6A—C5A—C4A	123.75 (11)	C12B—C7B—C6B	120.73 (11)
C6A—C5A—H5AA	118.1	C8B—C7B—C6B	122.07 (12)
C4A—C5A—H5AA	118.1	C9B—C8B—C7B	121.25 (13)
C5A—C6A—C7A	121.98 (10)	C9B—C8B—H8BA	119.4
C5A—C6A—C1A	119.92 (11)	C7B—C8B—H8BA	119.4
C7A—C6A—C1A	118.08 (10)	C8B—C9B—C10B	120.48 (12)
C12A—C7A—C8A	117.23 (11)	C8B—C9B—H9BA	119.8
C12A—C7A—C6A	121.58 (10)	C10B—C9B—H9BA	119.8
C8A—C7A—C6A	121.12 (10)	O2B—C10B—C11B	124.76 (13)
C9A—C8A—C7A	121.32 (11)	O2B—C10B—C9B	115.76 (12)
C9A—C8A—H8AA	119.3	C11B—C10B—C9B	119.48 (13)
C7A—C8A—H8AA	119.3	C12B—C11B—C10B	119.52 (12)
C8A—C9A—C10A	120.40 (11)	C12B—C11B—H11B	120.2
C8A—C9A—H9AA	119.8	C10B—C11B—H11B	120.2
C10A—C9A—H9AA	119.8	C11B—C12B—C7B	122.08 (12)
O2A—C10A—C11A	124.88 (11)	C11B—C12B—H12B	119.0
O2A—C10A—C9A	115.49 (10)	C7B—C12B—H12B	119.0
C11A—C10A—C9A	119.62 (11)	C18B—C13B—C14B	107.6 (8)
C10A—C11A—C12A	119.44 (11)	C18B—C13B—C2B	128.1 (5)
C10A—C11A—H11A	120.3	C14B—C13B—C2B	124.1 (5)
C12A—C11A—H11A	120.3	C15B—C14B—C13B	122.8 (5)
C11A—C12A—C7A	121.98 (11)	C15B—C14B—H14B	118.6
C11A—C12A—H12A	119.0	C13B—C14B—H14B	118.6
C7A—C12A—H12A	119.0	C16B—C15B—C14B	122.0 (5)
C18A—C13A—C14A	118.29 (12)	C16B—C15B—H15B	119.0
C18A—C13A—C2A	118.99 (12)	C14B—C15B—H15B	119.0
C14A—C13A—C2A	122.68 (12)	C15B—C16B—C17B	121.9 (7)
C13A—C14A—C15A	120.77 (13)	C15B—C16B—H16B	119.1
C13A—C14A—H14A	119.6	C17B—C16B—H16B	119.1
C15A—C14A—H14A	119.6	C18B—C17B—C16B	109.9 (9)
C16A—C15A—C14A	119.77 (13)	C18B—C17B—H17B	125.1
C16A—C15A—H15A	120.1	C16B—C17B—H17B	125.1
C14A—C15A—H15A	120.1	C17B—C18B—C13B	135.3 (10)
C17A—C16A—C15A	119.70 (12)	C17B—C18B—H18B	112.4
C17A—C16A—H16A	120.1	C13B—C18B—H18B	112.4
C15A—C16A—H16A	120.1	C18C—C13C—C14C	130.0 (11)
C16A—C17A—C18A	120.39 (14)	C18C—C13C—C2B	117.1 (7)
C16A—C17A—H17A	119.8	C14C—C13C—C2B	112.7 (7)
C18A—C17A—H17A	119.8	C13C—C14C—C15C	116.8 (7)
C13A—C18A—C17A	121.06 (13)	C13C—C14C—H14C	121.6
C13A—C18A—H18A	119.5	C15C—C14C—H14C	121.6
C17A—C18A—H18A	119.5	C16C—C15C—C14C	119.8 (6)
O3A—C19A—O1A	124.84 (13)	C16C—C15C—H15C	120.1
O3A—C19A—C3A	123.63 (14)	C14C—C15C—H15C	120.1
O1A—C19A—C3A	111.44 (11)	C15C—C16C—C17C	115.8 (8)
O1A—C20A—C21A	106.78 (15)	C15C—C16C—H16C	122.1
O1A—C20A—H20A	110.4	C17C—C16C—H16C	122.1
C21A—C20A—H20A	110.4	C16C—C17C—C18C	130.6 (11)
O1A—C20A—H20B	110.4	C16C—C17C—H17C	114.7
C21A—C20A—H20B	110.4	C18C—C17C—H17C	114.7
H20A—C20A—H20B	108.6	C13C—C18C—C17C	105.8 (11)
C20A—C21A—H21A	109.5	C13C—C18C—H18C	127.1
C20A—C21A—H21B	109.5	C17C—C18C—H18C	127.1
H21A—C21A—H21B	109.5	O3B—C19B—O1B	124.16 (14)
C20A—C21A—H21C	109.5	O3B—C19B—C3B	125.19 (14)
H21A—C21A—H21C	109.5	O1B—C19B—C3B	110.63 (12)
H21B—C21A—H21C	109.5	O1B—C20B—C21B	111.94 (14)
O2A—C22A—H22A	109.5	O1B—C20B—H20C	109.2
O2A—C22A—H22B	109.5	C21B—C20B—H20C	109.2
H22A—C22A—H22B	109.5	O1B—C20B—H20D	109.2
O2A—C22A—H22C	109.5	C21B—C20B—H20D	109.2
H22A—C22A—H22C	109.5	H20C—C20B—H20D	107.9
H22B—C22A—H22C	109.5	C20B—C21B—H21D	109.5
C19B—O1B—C20B	116.36 (12)	C20B—C21B—H21E	109.5
C10B—O2B—C22B	117.72 (11)	H21D—C21B—H21E	109.5
C6B—C1B—C2B	114.71 (10)	C20B—C21B—H21F	109.5
C6B—C1B—H1BA	108.6	H21D—C21B—H21F	109.5
C2B—C1B—H1BA	108.6	H21E—C21B—H21F	109.5
C6B—C1B—H1BB	108.6	O2B—C22B—H22D	109.5
C2B—C1B—H1BB	108.6	O2B—C22B—H22E	109.5
H1BA—C1B—H1BB	107.6	H22D—C22B—H22E	109.5
C13B—C2B—C1B	110.9 (3)	O2B—C22B—H22F	109.5
C13B—C2B—C3B	112.2 (3)	H22D—C22B—H22F	109.5
C1B—C2B—C3B	110.68 (11)	H22E—C22B—H22F	109.5
			
C6A—C1A—C2A—C13A	−178.56 (10)	C2B—C3B—C4B—O4B	−151.15 (14)
C6A—C1A—C2A—C3A	−54.04 (13)	C19B—C3B—C4B—C5B	153.85 (12)
C13A—C2A—C3A—C19A	−53.03 (14)	C2B—C3B—C4B—C5B	31.85 (17)
C1A—C2A—C3A—C19A	−179.27 (10)	O4B—C4B—C5B—C6B	176.25 (14)
C13A—C2A—C3A—C4A	−176.33 (10)	C3B—C4B—C5B—C6B	−6.8 (2)
C1A—C2A—C3A—C4A	57.43 (13)	C4B—C5B—C6B—C7B	179.42 (12)
C19A—C3A—C4A—O4A	26.66 (17)	C4B—C5B—C6B—C1B	1.78 (19)
C2A—C3A—C4A—O4A	149.73 (12)	C2B—C1B—C6B—C5B	−22.94 (17)
C19A—C3A—C4A—C5A	−156.21 (12)	C2B—C1B—C6B—C7B	159.32 (11)
C2A—C3A—C4A—C5A	−33.14 (15)	C5B—C6B—C7B—C12B	−157.01 (12)
O4A—C4A—C5A—C6A	−178.87 (13)	C1B—C6B—C7B—C12B	20.69 (17)
C3A—C4A—C5A—C6A	4.06 (19)	C5B—C6B—C7B—C8B	21.79 (19)
C4A—C5A—C6A—C7A	178.62 (11)	C1B—C6B—C7B—C8B	−160.50 (12)
C4A—C5A—C6A—C1A	0.20 (18)	C12B—C7B—C8B—C9B	0.1 (2)
C2A—C1A—C6A—C5A	25.66 (16)	C6B—C7B—C8B—C9B	−178.77 (13)
C2A—C1A—C6A—C7A	−152.81 (10)	C7B—C8B—C9B—C10B	0.3 (2)
C5A—C6A—C7A—C12A	167.45 (12)	C22B—O2B—C10B—C11B	4.9 (2)
C1A—C6A—C7A—C12A	−14.11 (16)	C22B—O2B—C10B—C9B	−175.43 (12)
C5A—C6A—C7A—C8A	−15.68 (18)	C8B—C9B—C10B—O2B	179.92 (12)
C1A—C6A—C7A—C8A	162.76 (11)	C8B—C9B—C10B—C11B	−0.4 (2)
C12A—C7A—C8A—C9A	0.45 (18)	O2B—C10B—C11B—C12B	179.76 (12)
C6A—C7A—C8A—C9A	−176.55 (11)	C9B—C10B—C11B—C12B	0.1 (2)
C7A—C8A—C9A—C10A	0.29 (19)	C10B—C11B—C12B—C7B	0.3 (2)
C22A—O2A—C10A—C11A	4.94 (17)	C8B—C7B—C12B—C11B	−0.37 (19)
C22A—O2A—C10A—C9A	−174.41 (11)	C6B—C7B—C12B—C11B	178.49 (12)
C8A—C9A—C10A—O2A	178.32 (11)	C1B—C2B—C13B—C18B	−52.5 (10)
C8A—C9A—C10A—C11A	−1.07 (18)	C3B—C2B—C13B—C18B	71.8 (9)
O2A—C10A—C11A—C12A	−178.25 (11)	C13C—C2B—C13B—C18B	65 (6)
C9A—C10A—C11A—C12A	1.08 (18)	C1B—C2B—C13B—C14B	121.2 (6)
C10A—C11A—C12A—C7A	−0.33 (19)	C3B—C2B—C13B—C14B	−114.5 (6)
C8A—C7A—C12A—C11A	−0.43 (18)	C13C—C2B—C13B—C14B	−122 (7)
C6A—C7A—C12A—C11A	176.55 (11)	C18B—C13B—C14B—C15B	−3.5 (9)
C1A—C2A—C13A—C18A	−122.41 (13)	C2B—C13B—C14B—C15B	−178.3 (5)
C3A—C2A—C13A—C18A	113.87 (14)	C13B—C14B—C15B—C16B	0.2 (7)
C1A—C2A—C13A—C14A	60.06 (16)	C14B—C15B—C16B—C17B	−0.6 (9)
C3A—C2A—C13A—C14A	−63.66 (16)	C15B—C16B—C17B—C18B	4.2 (13)
C18A—C13A—C14A—C15A	1.1 (2)	C16B—C17B—C18B—C13B	−10 (2)
C2A—C13A—C14A—C15A	178.63 (12)	C14B—C13B—C18B—C17B	9.5 (19)
C13A—C14A—C15A—C16A	−0.2 (2)	C2B—C13B—C18B—C17B	−176.0 (12)
C14A—C15A—C16A—C17A	−0.8 (2)	C13B—C2B—C13C—C18C	−111 (7)
C15A—C16A—C17A—C18A	0.9 (2)	C1B—C2B—C13C—C18C	−46.3 (10)
C14A—C13A—C18A—C17A	−1.0 (2)	C3B—C2B—C13C—C18C	76.2 (9)
C2A—C13A—C18A—C17A	−178.61 (13)	C13B—C2B—C13C—C14C	73 (6)
C16A—C17A—C18A—C13A	0.0 (2)	C1B—C2B—C13C—C14C	137.9 (6)
C20A—O1A—C19A—O3A	−4.8 (2)	C3B—C2B—C13C—C14C	−99.7 (7)
C20A—O1A—C19A—C3A	171.95 (12)	C18C—C13C—C14C—C15C	4.7 (13)
C4A—C3A—C19A—O3A	−119.90 (14)	C2B—C13C—C14C—C15C	179.9 (5)
C2A—C3A—C19A—O3A	117.39 (14)	C13C—C14C—C15C—C16C	2.1 (8)
C4A—C3A—C19A—O1A	63.32 (14)	C14C—C15C—C16C—C17C	−0.3 (11)
C2A—C3A—C19A—O1A	−59.39 (14)	C15C—C16C—C17C—C18C	−8.9 (19)
C19A—O1A—C20A—C21A	−169.57 (14)	C14C—C13C—C18C—C17C	−11.1 (17)
C6B—C1B—C2B—C13B	172.3 (3)	C2B—C13C—C18C—C17C	173.9 (9)
C6B—C1B—C2B—C3B	47.14 (15)	C16C—C17C—C18C—C13C	13 (2)
C6B—C1B—C2B—C13C	167.8 (4)	C20B—O1B—C19B—O3B	4.1 (2)
C13B—C2B—C3B—C19B	65.7 (2)	C20B—O1B—C19B—C3B	−174.31 (12)
C1B—C2B—C3B—C19B	−169.88 (11)	C4B—C3B—C19B—O3B	−73.10 (17)
C13C—C2B—C3B—C19B	66.3 (3)	C2B—C3B—C19B—O3B	48.92 (19)
C13B—C2B—C3B—C4B	−175.3 (2)	C4B—C3B—C19B—O1B	105.25 (13)
C1B—C2B—C3B—C4B	−50.81 (14)	C2B—C3B—C19B—O1B	−132.73 (12)
C13C—C2B—C3B—C4B	−174.7 (3)	C19B—O1B—C20B—C21B	−80.67 (19)
C19B—C3B—C4B—O4B	−29.15 (18)		

### Hydrogen-bond geometry (Å, °)

**Table:**

*D*—H···*A*	*D*—H	H···*A*	*D*···*A*	*D*—H···*A*
C3A—H3AA···O2B^i^	1.00	2.57	3.4093 (18)	142
C5A—H5AA···O4B^ii^	0.95	2.55	3.3630 (17)	143
C8A—H8AA···O4B^ii^	0.95	2.31	3.2446 (18)	166
C14B—H14B···O2A^i^	0.95	2.58	3.500 (5)	163
C16A—H16A···O2B^iii^	0.95	2.47	3.2789 (18)	143
C17B—H17B···O3A^iv^	0.95	2.47	3.116 (9)	125
C21B—H21D···Cg1^v^	0.98	2.85	3.811 (2)	167
C22B—H22D···Cg2^vi^	0.98	2.87	3.644 (4)	137

Symmetry codes: (i) −*x*, −*y*+1, −*z*+2; (ii) −*x*+1, −*y*+1, −*z*+2; (iii) *x*, *y*+1, *z*−1; (iv) *x*, *y*−1, *z*; (v) *x*+1, *y*, *z*; (vi) −*x*, −*y*, −*z*+2.
